# P-1633. Evaluating an Urgent Care Antibiotic Stewardship Intervention: A Multi-Network Collaborative Effort

**DOI:** 10.1093/ofid/ofae631.1799

**Published:** 2025-01-29

**Authors:** Annie Roberts, Daniel E Park, Sabrina Balthrop, Rana F Hamdy, Rana F Hamdy, Patrick Dolan, Cindy Liu

**Affiliations:** The George Washington University, Washington, District of Columbia; The George Washington University, Washington, District of Columbia; Urgent Care Association, Batavia, Illinois; Childrens National Hospital, Washington, District of Columbia; Childrens National Hospital, Washington, District of Columbia; PM Pediatrics, Mount Prospect, Illinois; George Washington University Milken School of Public Health, Washington, DC

## Abstract

**Background:**

Urgent care centers (UCCs) have previously been associated with high rates of inappropriate antibiotic prescribing without supporting appropriate diagnoses. Prior antibiotic stewardship studies in urgent care settings have generally been limited to pediatric clinics and diagnoses or conducted within single urgent care networks. Broadly generalizable stewardship efforts targeting common diagnoses across ages are needed.

**Figure:**
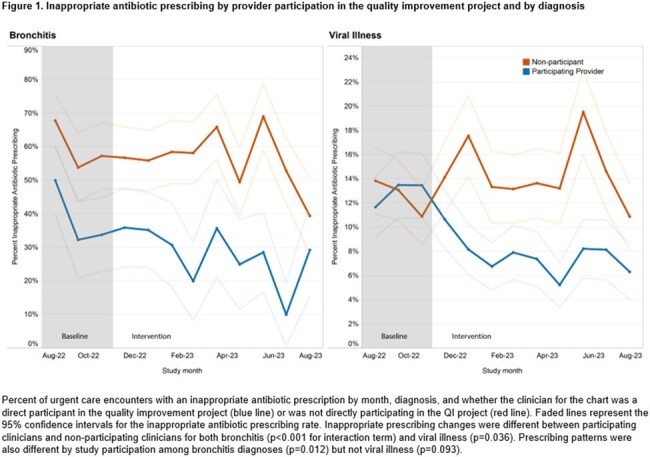

**Methods:**

This quality improvement study's participants were UCC clinicians from a national collaborative of 49 UCCs in 27 networks across 18 states within the United States. Stewardship interventions included signing of a commitment statement, and a choice of intervention options to implement during two plan-do-study-act (PDSA) cycles during the intervention period. The primary outcome was the percent of urgent care encounters (from randomly selected patient charts) for viral illness or bronchitis diagnoses with inappropriate antibiotic prescribing, stratified by whether the clinician was a direct participant in the quality improvement study and by diagnosis. A 3-month baseline and 9-month intervention period were compared using an interrupted time series with a generalized estimating equation model.

**Figure:**
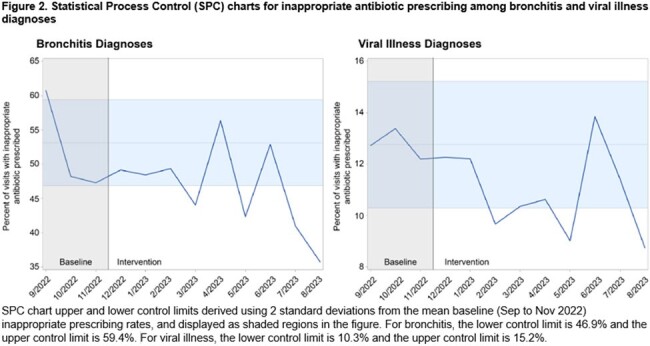

**Results:**

15,588 patient encounters were included with a diagnosis of bronchitis or viral illness. The intervention was associated with a 39% relative decrease in inappropriate antibiotic prescribing (aOR=0.61, 95%CI 0.48-0.77) among participating clinicians compared to baseline. The intervention did not result in a significant change in inappropriate antibiotic prescribing (aOR=1.08, p=0.54) for clinicians who did not directly participate.

**Figure:**
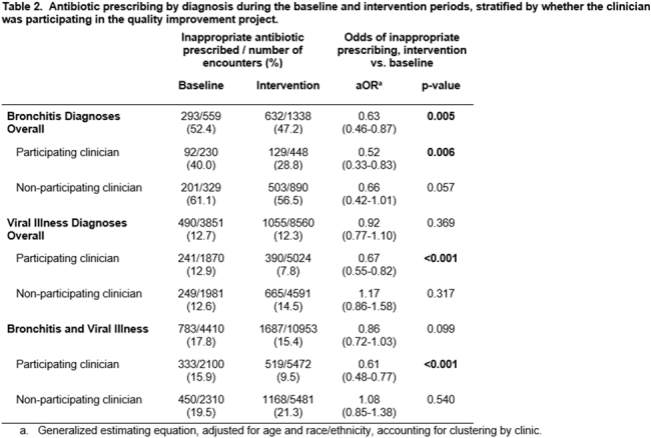

**Conclusion:**

This antibiotic stewardship intervention was associated with significant reductions in inappropriate prescribing among clinicians who directly participated. Implementing stewardship interventions in UCCs may reduce inappropriate antibiotic prescriptions for common diagnoses; however, direct clinician participation may be necessary, especially in settings with high rates of clinician turnover.

**Disclosures:**

**All Authors**: No reported disclosures

